# Behavioral Analyses in Dark Agouti Rats Following Repeated Systemic Treatment With Fingolimod (FTY720)

**DOI:** 10.1002/brb3.70146

**Published:** 2024-11-17

**Authors:** Marie Jakobs, Lisa Trautmann, Martin Hadamitzky, Julia Bihorac, Lucie Jacquet, Uwe Christians, Björn Schniedewind, Laura Lückemann, Manfred Schedlowski

**Affiliations:** ^1^ Institute of Medical Psychology and Behavioral Immunobiology, Center for Translational Neuro‐Behavioral Sciences (C‐TNBS) University Medicine Essen, University Duisburg‐Essen Essen Germany; ^2^ Department of Infectious Diseases, West German Centre of Infectious Diseases University Medicine Essen, University Duisburg‐Essen Essen Germany; ^3^ iC42 Clinical Research and Development, Department of Anesthesiology, School of Medicine University of Colorado Anschutz Medical Campus Aurora Colorado USA; ^4^ Department of Clinical Neuroscience Osher Center for Integrative Medicine, Karolinska Institutet Stockholm Sweden

**Keywords:** adverse side effects, anxiety‐like behavior, fingolimod (FTY720), immunosuppressive drugs

## Abstract

**Background:**

Studies in experimental animals revealed that acute and chronic treatment with small‐molecule immunosuppressive drugs lead to neurobehavioral alterations in rodents.

**Methods:**

Against this background, this study investigated behavioral alterations in rats after repeated administration of FTY720, an immunosuppressive drug used for the treatment of multiple sclerosis, employing the open field, elevated plus maze, and dark/light tests.

**Results:**

Compared to controls, repeated FTY720 treatment affected behavior in rats, reflected by a reduction in distance traveled as well as increased time engaged in freezing in the open field and elevated plus maze. Furthermore, the time spent freezing in the elevated plus maze test positively correlated with FTY720 concentrations in the amygdala and insular cortex, two brain regions involved in regulation of emotionality. Since no changes in plasma corticosterone levels were observed, stress effects due to treatment, behavioral testing, or handling can be ruled out.

**Conclusion:**

The present findings indicate that treatment with FTY720 did not induce typical anxiety‐like behavioral patterns in otherwise healthy rats as seen following treatment with other immunosuppressive drugs. Nevertheless, it remains of great importance to evaluate behavioral effects in clinical practice to shed more light onto possible detrimental side effects emerging during treatment with small‐molecule immunosuppressive drugs.

## Graphical abstract

Peripheral administered FTY720 accumulates in the insular cortex and amygdala and induces anxiety‐like behavior in dark agouti rats (created with BioRender.com).

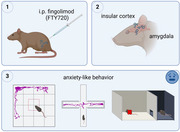

## 1 Introduction

Various experimental studies demonstrated neuropsychological alterations during treatment with small‐molecule immunosuppressive drugs (SMDs) in otherwise healthy animals (Bosche et al. 2015; Mason 1990). More specific, acute and chronic systemic administration of calcineurin inhibitors, such as cyclosporine A (CsA) and tacrolimus (FK506), have been shown to result in elevated levels of anxiety‐like behavior (von Horsten et al. 1998), most probably due to drug‐induced blockade of calcineurin activity in the amygdala (Mineur, Taylor, and Picciotto 2014). Acute systemic administration of CsA also caused increased neuronal activity and c‐FOS expression in the amygdala (Pacheco‐Lopez et al. 2013), abnormalities considered as high‐risk factors for the development of fear and anxiety disorders (Wolfensberger et al. 2008). Likewise, high‐dose treatment with CsA affected neurotransmitter release and increased anxiety‐like and social behavior in mice (Sato et al. 2007). When chronically injected into the medial prefrontal cortex (mPFC) of rats, FK506 even increased depressive‐like behavior, which could be reversed by *N*‐methyl‐d‐aspartate (NMDA) or the selective noradrenalin re‐uptake inhibitor venlafaxine (Yu et al. 2013).

Similar results have also been found in rodents treated with mechanistic target of rapamycin (mTOR) inhibitors, such as rapaymcin or temsirolimus. In this context, a pattern of anxiety‐related behavior emerged not only during acute (Hadamitzky et al. 2014; Tsai et al. 2013) but also following chronic administration of mTOR inhibitors (Lu et al. 2015).

Fingolimod (FTY720), a sphingosine 1‐phosphate (S1P) receptor agonist, is widely used for the treatment of multiple sclerosis. Its primary immunological action is characterized by preventing lymphocyte egress from secondary lymphoid organs, resulting in significant lymphopenia in peripheral blood (Brinkmann et al. 2010). FTY720 can also cross the blood–brain barrier (BBB) (Cipriani et al. 2017), thereby interacting with oligodendrocytes (Roggeri et al. 2020) and inducing neuroprotective effects (Ambrosius et al. 2017; Bascuñana et al. 2020). Importantly, FTY720 has been shown to be an effective compound for eliminating aversive memories in diseases such as post‐traumatic stress disorder (Hait et al. 2014). However, reports regarding adverse effects of this drug on brain and behavior are very sparse.

To bridge this important gap and to extend the knowledge regarding behavioral alterations following treatment with immunosuppressive drugs, this study explored the impact of repeated systemic FTY720 administration (1 mg/kg bw) on behavioral performance with a focus on anxiety‐like behavior in healthy male dark agouti (DA) rats, using the open field (OF), the elevated plus maze (EPM), and the dark/light tests. Additionally, FTY720 concentrations were measured in the plasma, in the insular cortex (IC), and in the amygdala (AM), two brain regions implicated in the regulation of emotionality (Dantzer et al. 2008) and taste‐immune associative learning (Hadamitzky et al. 2020).

## 2 Materials and Methods

### 2.1 Animals

In total, 24 male DA (HanRj) rats (215–246 g; Janvier, France) were used in this experiment. Animals were set on an inverse 12 h light/dark cycle (lights off at 7 a.m.), single‐housed with ad libitum access to food and tap water and handled daily for 1 week prior to and during the experiments. The animal facilities and experimental procedures were in accordance with the ARRIVE guidelines (Percie du Sert et al. 2020) as well as the National Institutes of Health and Association for the Assessment and Accreditation of Laboratory Animal Care guidelines and were approved by the Institutional Animal Care and Use Committee (LANUV Düsseldorf, North Rhine‐Westphalia, G1806/20, Az. 81‐02.04.2020.A322).

### 2.2 Drug Administration

The used FTY720 dose was chosen based on former studies (Jakobs et al. 2024; Serdar et al. 2016). FTY720 (Sigma‐Aldrich) was dissolved in 0.9% NaCl (Berlin‐Chemie AG). FTY720 stock solution (1 mg/mL) vials and control NaCl vials were stored at 4°C. Solutions were injected at room temperature (RT). Animals were randomly assigned to the experimental group (*n* = 12), which was i.p. injected three times every 72 h with 1 mg/mL FTY720 and to the control group (*n* = 12), which was i.p. injected with 0.9% saline following the same scheme. Each animal was injected with an injection volume of 1 mL/kg bw.

### 2.3 Plasma Corticosterone Analysis

After behavioral testing, tail vein blood was collected in EDTA monovettes (Sarstedt) under isoflurane anesthesia. Plasma was separated by centrifugation (2000 × *g*, 10 min, 4°C) and stored at −80°C until further processing. Plasma corticosterone concentrations were determined using a commercial corticosterone ELISA (IBL International) according to manufacturer's instructions.

### 2.4 FTY720 Quantification

At the end of the experiments, blood was drawn under isoflurane anesthesia before animals were sacrificed by decapitation. Plasma was separated by centrifugation (2000 × *g*, 10 min, 4°C) and stored at −80°C until further processed. Brains were quickly removed, frozen in isopentan and dry ice, and stored at −80°C. Using a cryostat microtome (CM1950, Leica), 200‐µm thick coronal brain sections were cut at −18°C and transferred to prechilled glass slides. The AM and IC were then dissected from five serial brain sections using a micropunch technique (Cuello and Carson 1983; Lückemann et al. 2021). Briefly, a prechilled stainless steel sample puncher (Ø 2 mm; Fine Science Tools) was used to isolate tissue samples of the left and right IC and AM. Optical tract and hippocampus served as anatomical landmarks to ensure comparable positions of the punched samples across animals (Paxinos and Watson 1998). FTY720 concentrations in plasma and brain tissue were analyzed as described previously with a few modifications (Gottschalk et al. 2011). Briefly, brain samples were weighed and homogenized with phosphate‐buffered saline (pH = 7.4). After protein precipitation with 30% 0.2 M ZnSO_4_ in water/70% methanol containing the internal standard fingolimod D_4_, samples were vortexed for 2.5 min and centrifuged (16,000 × *g*, 10 min). Twenty‐five‐microliter supernatants were injected into a 2D high‐performance liquid chromatography‐tandem mass spectrometry (LC‐MS/MS) system for further online extraction and quantification of FTY720 (for details, see Supporting Information).

### 2.5 Behavioral Analyses

All behavioral testing were performed during the activity period of the animals (dark phase) under red‐light illumination by the experimentators' blind. Prior to testing, rats were transferred to the experimental room and were allowed to habituate for 1 h. Between the trials, test apparatuses were cleaned with 70% ethanol to eliminate possible odor cues of previous animals. Prior to FTY720 administration, baseline levels for each test were recorded. Subsequently, animals were randomly assigned to the FTY720‐treated experimental and saline‐treated control groups. On the testing day, behavioral tasks were conducted 1 h following FTY720 administration.

#### 2.5.1 Open‐Field Test

The OF apparatus (75 × 75 × 50 cm) comprised four separated enclosed chambers of the same size. Testing started by individually placing the animals in the center of one chamber, and performance was assessed over a testing period of 15 min, using an automatized video tracking system (Stoelting; ANY‐maze 7.09). Parameters analyzed were the total distance traveled, the average speed, the freezing time, and the number of entries into the inner zone (25 × 25 cm) of the arena.

#### 2.5.2 Elevated Plus Maze

The EPM apparatus comprised a central section (15 × 15 cm), two opposing open arms (42.5 × 15 cm), and two opposing closed arms (42.5 × 15 × 22.5 cm). Rats were placed into the central section facing a closed arm. The total distance traveled, the average speed, the freezing time (suppression of all movement except that required for respiration), and the time spent in the open arms were recorded for 5 min using an automatized video tracking system (Stoelting; ANY‐maze 7.09).

#### 2.5.3 Dark/Light Test

The dark/light test was conducted in a box (80 × 35 × 35 cm) divided into a dark compartment (35 × 35 × 35 cm) and a bright compartment (45 × 35 × 35 cm). Both chambers were connected via an open door. Rats were placed into the dark compartment and were allowed to move freely between the two chambers. The number of transitions into the bright compartment (whole body) and the number of times the rats protruded their heads into the bright chamber were assessed manually for 15 min.

### 2.6 Statistical Analysis

Behavioral experiments were replicated with *n* = 6 animals in each group (experimental and control). Statistical analyses were performed with GraphPad Prism (Version 8) and the level of significance was set at *p* < 0.05. The normality of residuals was examined using the D'Agostino and Pearson test. Subsequently, behavioral data and plasma cortisol concentrations were analyzed using a two‐tailed *t* test with Welch's correction. To assess whether and to what extend behavioral data as well as plasma corticosterone concentrations correlate with FTY720 tissue concentrations, Spearman correlation analysis was applied. Merged from two independent experiments data were evaluated as mean percentage changes from baseline.

## 3 Results

### 3.1 FTY720 Quantification

Quantification of FTY720 in plasma and brain regions (AM, IC) using LC‐MS/MS verified measurable concentrations of FTY720 in these tissues following acute i.p. administration of 1 mg/kg bw FTY720 three times every 72 h. FTY720 plasma concentrations (ranged from 10.8 to 18.7 ng/mL (mean = 14.93, SEM = 0.69), while FTY720 tissue concentrations in the AM ranged from 6.52 to 19.6 ng/mg (mean = 13.50, SEM = 1.13) and in the IC from 10 to 21.8 ng/mg (mean = 15.85, SEM = 1.1).

### 3.2 Behavioral Measurements

**FIGURE 1:**
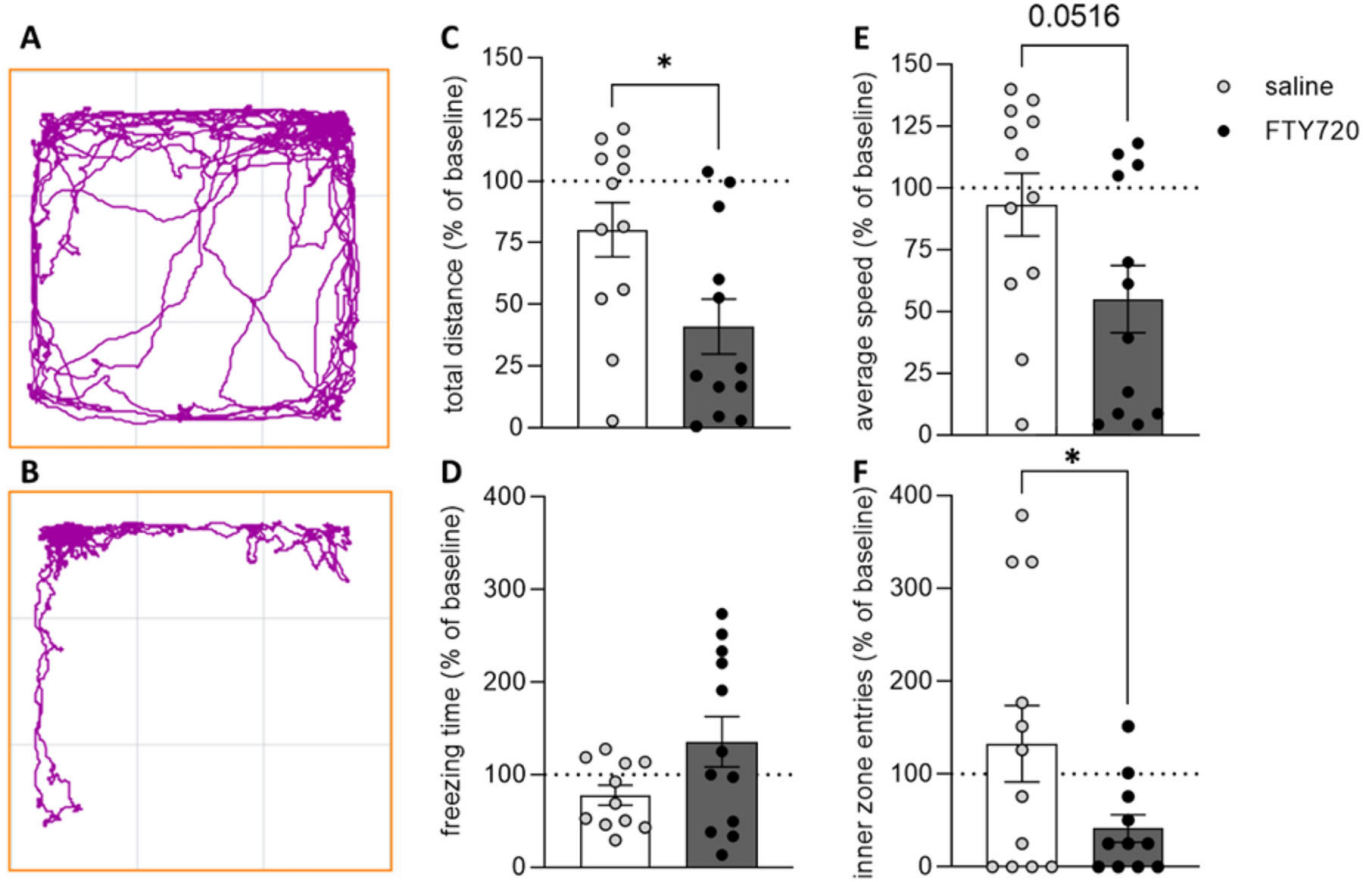
Effect of FTY720 on anxiety behavior assessed using the OF test. Exemplary track plots illustrate the distance traveled by rats injected three times every 72 h with either saline (A) or 1 mg/kg bw FTY720 (B) during the 15‐min OF test. Treatment with FTY720 resulted in a reduction of the total distance traveled (C), the average speed (E), and the number of entries into the inner zone (F) compared to saline‐treated animals. FTY720 treatment did not affect the freezing time (D). Asterisks indicate a statistically significant difference between groups (two‐tailed *t* test; *p < 0.05; saline = control group (*n* = 11–12); FTY720 = pharmacological group (*n* = 11–12). Results are presented as mean percentage changes normalized to baseline levels ± SEM.

Animals treated with FTY720 displayed a significant reduction in the total distance traveled (*p* = 0.020), the average speed (*p* = 0.052), and the number of entries into the inner zone (*p* = 0.026) compared to saline‐treated animals (Figure 1).

**FIGURE 2:**
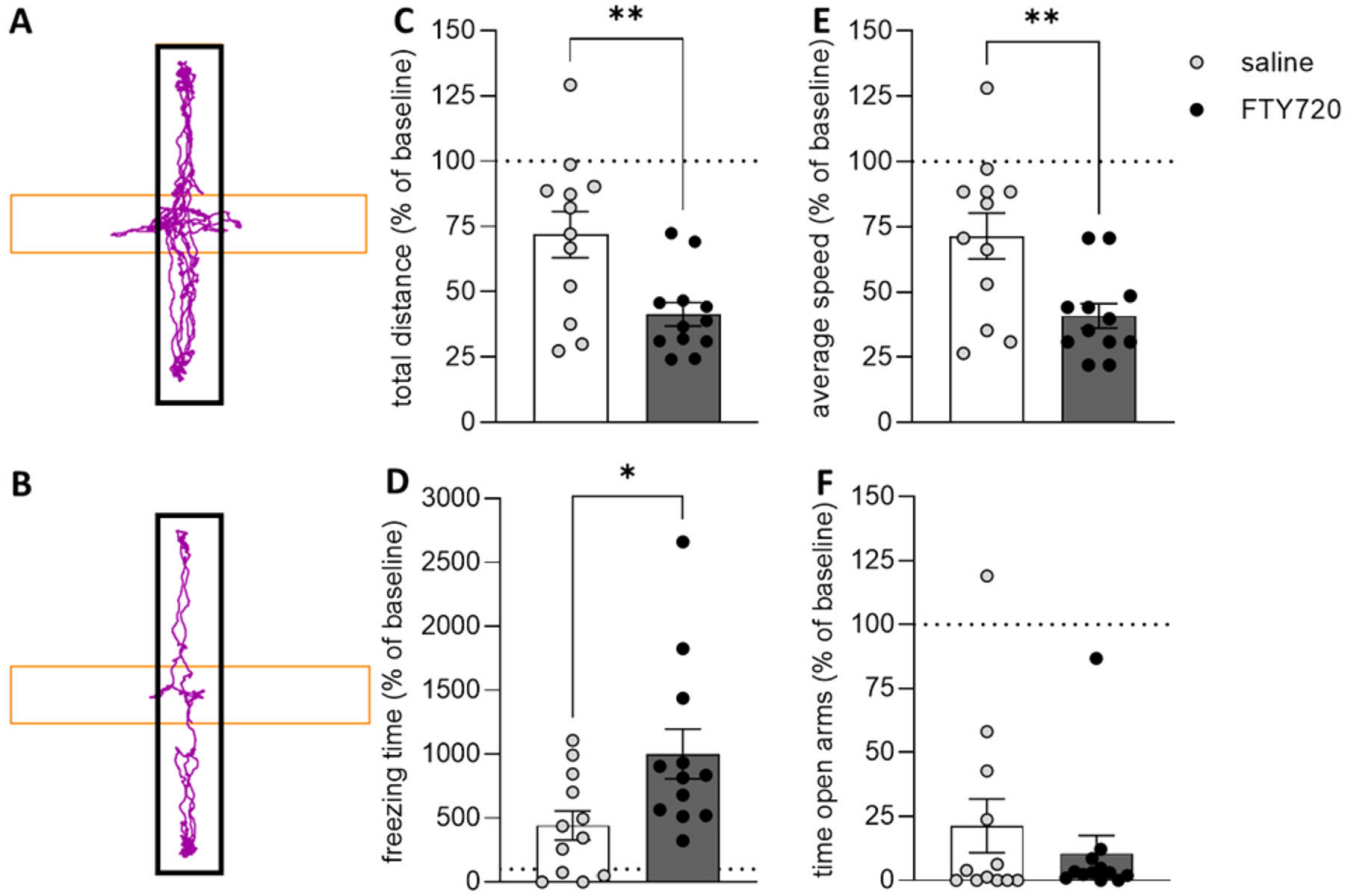
Effect of FTY720 on anxiety behavior assessed using the EPM test. Exemplary track plots illustrate the distance that rats, injected three times every 72 h with either saline (A) or 1 mg/kg bw FTY720 (B), traveled during the 5‐min EPM test. Black borders indicate the closed arms. FTY720 treatment led to a reduction in the total distance traveled (C), the average speed (E), and an increase in the time spent freezing (D) compared to saline‐treated animals. In contrast, FTY720 treatment did not affect the time spent in the open arms (F) compared to saline‐treated rats. Asterisks indicate a statistically significant difference between groups (two‐tailed *t* test; *p < 0.05, **p < 0.01; saline = control group (*n* = 12); FTY720 = pharmacological group (*n* = 12). Results are presented as mean percentage changes normalized to baseline levels ± SEM.

Additionally, repeated administration of FTY720 resulted in a statistically significant reduction in both the total distance traveled (*p* = 0.007) and average speed (*p* = 0.007) on the EPM test. Compared to controls, FTY720‐treated rats also showed a significant increase in the total time spent freezing on the maze (*p* = 0.045; Figure 2).

**FIGURE 3:**
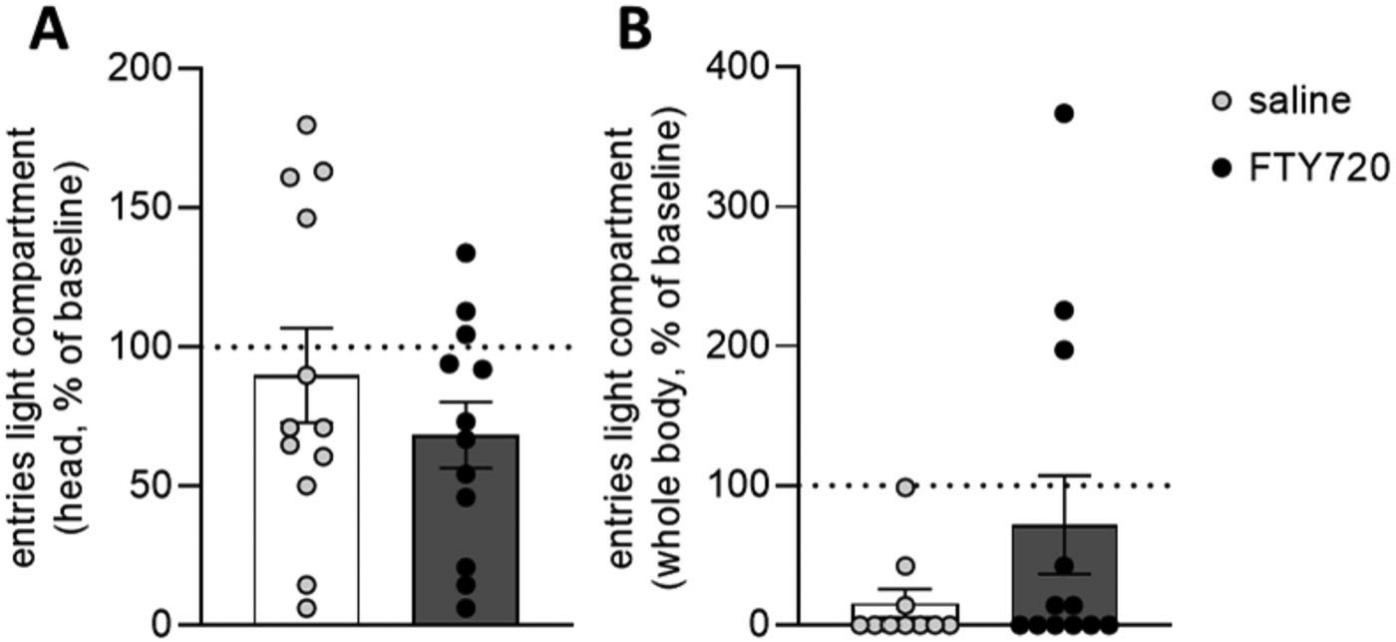
Effect of FTY720 on anxiety behavior assessed using the dark/light test. FTY720 treatment (1 mg/kg bw, three times every 72 h) did not affect the number of times rats entered the light compartment with either their head (A) or whole body (B) compared to saline‐treated rats. Saline = control group (*n* = 10–12); FTY720 = pharmacological group (*n* = 12). Results are presented as mean percentage changes normalized to baseline levels ± SEM.

Employing the dark/light test, no significant differences between FTY720‐treated rats and controls were observed (Figure 3).

### 3.3 Physiological Measurements

**FIGURE 4:**
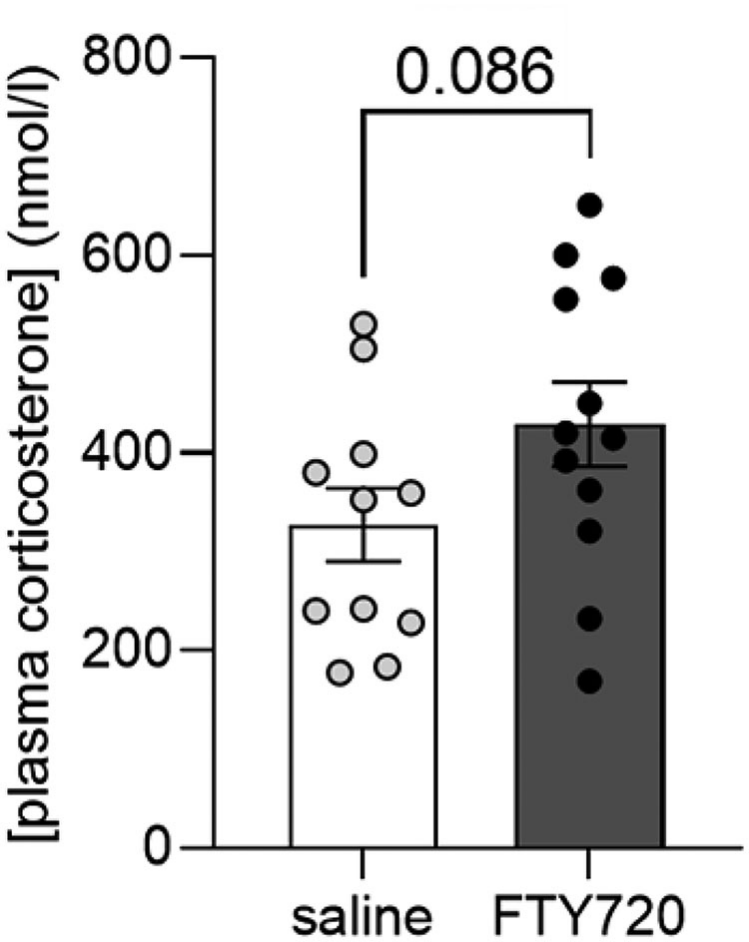
Effect of FTY720 on plasma corticosterone concentrations. FTY720 treatment (1 mg/kg bw, three times every 72 h) did not affect plasma corticosterone concentrations compared to saline‐treated rats. Saline = control group (*n* = 12); FTY720 = pharmacological group (*n* = 12). Results are presented as mean ± SEM.

Steroid hormone analysis revealed that plasma corticosterone concentrations did not differ between repeatedly FTY720‐treated animals and controls (*p* = 0.086; Figure 4).

### 3.4 Correlation of Behavioral Pattern and Central FTY720 Tissue Concentrations

Analyzing the relationship between behavior and the FTY720 tissue concentrations, we found a statistically significant positive correlation (*p* = 0.02, *r* = 0.66) between the time animals spent freezing in the EPM and the drug concentrations in the AM (Figure S2B). A negative correlation (*p* = 0.06, *r* = −0.57) between the total distance traveled during the EPM and the FTY720 concentration in the AM (Figure S2A) as well for a negative correlation (*p* = 0.07, *r* = −0.54) between the time rats spent in the open arms in the EPM and FTY720 concentration in the IC did not reach level of significance (Figure S2H).

## 4 Discussion

This study aimed at investigating potential adverse effects of repeated systemic FTY720 administration (1 mg/kg bw) in healthy male DA rats, focusing on anxiety‐like behavior. Although no anxiety‐like behavior was observed, the data show that repeated FTY720 treatment affected performance in both the OF and the EPM. This was reflected by a reduction in the distance traveled, diminished average speed, and an increase in the time animals engaged freezing. Moreover, FTY720 was detectable in the AM, where drug concentrations correlated with the observed freezing behavior.

Previous experimental studies have demonstrated that both acute and chronic administration of SMDs (i.e., CsA, rapamycin) resulted in neurobehavioral changes in otherwise healthy animals (Brosda et al. 2021; Hadamitzky et al. 2014, 2018; von Horsten et al. 1998). However, the present findings do not align with these observations, since repeated FTY720 treatment did not affect relevant measures of anxiety‐like behaviors neither in the EPM test nor in the OF test. In general, drug‐ or stress‐induced anxiety‐like behavior assessed on the EPM is commonly reflected by a reduced number of entries into the open arms, and the increased time spent on the closed arms of the maze compared to controls (Crawley 1999; Enkel, Thomas, and Bartsch 2013; Gray 1979; Pellow et al. 1985). In this study, none of these “valid” markers for anxiety‐like behavior differed between the treatment and the control group (Figure 2). Avoidance behavior of rats to open spaces (Gentsch, Lichtsteiner, and Feer 1987) can be assessed in the OF, where the time the animal spends in the center of the arena is considered as index for anxiety‐like behavior. Nevertheless, this behavioral pattern was also not altered in FTY720‐treated animals (Figure 1). The absence of behavioral alterations in the dark/light test is somewhat not surprising (Figure 3). Considering that the dark/light test was the last in the series of behavioral tests, repeated testing of animals across different behavioral assessments could have already influenced behavior or interfered with subsequent tests (Lad et al. 2010; McIlwain et al. 2001).

In both tests, the EPM and the OF, we observed a rather robust impact on overall locomotor activity, which was accompanied by increased time FTY720‐treated animals engaged in freezing. Although not referred to as a critical component of anxiety‐like behavior, it is named as behavioral element of anxiety‐like behavior, at least in the OF test (Sestakova et al. 2013). Another possible explanation for the observed behavior may be the possible emergence of sedation due to FTY720 treatment. However, we cannot verify this hypothesis, since we did not apply a score rating on sedation, which includes additional measures such as righting reflex, eyes partly closed, or a downward head (Andersen et al. 2022). The finding that closed arm entries, which are considered as an indicator of locomotion (McCormick, Smith, and Mathews 2008), did not differ between FTY720‐treated animals and controls (data not shown) does not robustly support this hypothesis.

Importantly, acute FTY720 administration had no statistically significant impact on plasma corticosterone levels (Figure 4), suggesting that the observed behavioral alterations are not due to stress and increased hypothalamic‐pituitary‐adrenal (HPA) axis activity. These findings are consistent with those of a study examining the impact of the SMD rapamycin on anxiety‐like behavior. In that case, rapamycin induced a phenotype resembling anxiety‐like symptoms without affecting plasma corticosterone levels (Hadamitzky et al. 2018). In contrast, it has been reported that a higher dose of peripheral administered FTY720 (2.5 mg/kg bw) led to an increase of ACTH and corticosterone plasma concentrations when blood was drawn from the tail vein of restrained rats 1 h after the injection (Corbett et al. 2021). However, this effect disappeared after being restrained for 15 min, indicating that FTY720 treatment had no effect on HPA axis activity compared to saline‐treated animals during persistent stress.

Previous animal studies have shown that both acute and subchronic treatment with the immunosuppressive calcineurin inhibitor CsA affect behavior. Specifically, it impacted prepulse inhibition (PPI) of the acoustic startle response and led to a reduction in tyrosine hydroxylase activity in the prefrontal cortex. This suggests a decrease in dopamine synthesis and indicates the influence of the drug on the dopaminergic neurotransmitter system (Brosda et al. 2021). However, whether FTY720 also exerts a direct or an indirect effect on neurotransmitter systems, thereby inducing the here observed alterations on locomotor activity/freezing remains to be investigated.

Notably, a “disease‐dependent” antidepressant‐like potential of FTY720 through chronic treatment has been confirmed in MS patients (Montalban et al. 2011) and in mice subjected to chronic stress (di Nuzzo et al. 2015). However, continuous treatment with FTY720 in mice under chronic stress did not improve anxiety‐like behavior in the EPM test (di Nuzzo et al. 2015), underscoring the complexity of neuro‐immune interactions.

Regardless of the mechanism, the ability of drugs to cross the blood–brain barrier appears essential for influencing behavior and cognition (Ho et al. 2021). FTY720 has already been shown to cross the blood–brain barrier and to accumulate in specific brain regions (Jakobs et al. 2024). We here report a positive correlation (Figure S2) of the time animals spent freezing on the EPM and the FTY720 concentration in the AM (Figure S2)—a limbic region in the medial temporal lobe considered to be a central element in mood regulation, anxiety in particular (Dantzer et al. 2008).

However, the detection of FTY720 in the AM does not conclusively indicate that FTY720 activates this brain region. A significant limitation of this study is the lack of assessing the expression of neuronal activation markers (e.g., c‐FOS) (Hadamitzky et al. 2014) or the expression of proteins associated with the development of stress‐induced anxiety‐like behavior (e.g., FKBP51, KLK8, glucocorticoid receptor) (Attwood et al. 2011; Hadamitzky et al. 2018; Schmidt et al. 2012). Thus, it could be argued that FTY720 merely impairs locomotor activity rather than affecting anxiety‐like behavior. However, other studies have reported that FTY720 does not impact locomotor activity in wild type animals (Di Pardo et al. 2014; Li et al. 2023) or in disease conditions (Serdar et al. 2016).

## 5 Conclusion

In summary, the current data indicate that the immunosuppressive drug FTY720 somewhat affects behavior in healthy rats without clear changes on valid measures of induced anxiety‐like behavior. These findings underscore the importance of considering possible adverse side effects (i.e., those affecting the CNS) of immunomodulating drugs and highlight the necessity of further investigating the mechanisms of neuro‐immune interaction.

## Author Contributions

**Marie Jakobs**: Conceptualization, investigation, writing–original draft, methodology, visualization; writing–review and editing. **Lisa Trautmann**: methodology, investigation, software. **Martin Hadamitzky**: Conceptualization, funding acquisition, writing–review and editing. **Julia Bihorac**: Methodology. **Lucie Jacquet**: Methodology. **Uwe Christians**: Methodology. **Björn Schniedewind**: Methodology. **Laura Lückemann**: Methodology, investigation, supervision, conceptualization. **Manfred Schedlowski**: Funding acquisition, writing–review and editing.

## Conflicts of Interest

The authors declare no conflicts of interest.

### Peer Review

The peer review history for this article is available at https://publons.com/publon/10.1002/brb3.70146.

## Supporting information



Supporting Information

Supporting Information

## Data Availability

The datasets generated and analyzed during the current study are available from the corresponding author upon reasonable request.
