# Supplementary material for: Behavioral Analyses in Dark Agouti Rats Following Repeated Systemic Treatment With Fingolimod (FTY720)
Source: Brain Behav. 2024 Nov 17;14(11):e70146. doi: 10.1002/brb3.70146 (PMC11570679; doi:10.1002/brb3.70146)
Supplement: Supplementary file 1 — Supporting Information [file BRB3-14-e70146-s001.pdf]

Supplementary data

Supplementary figures

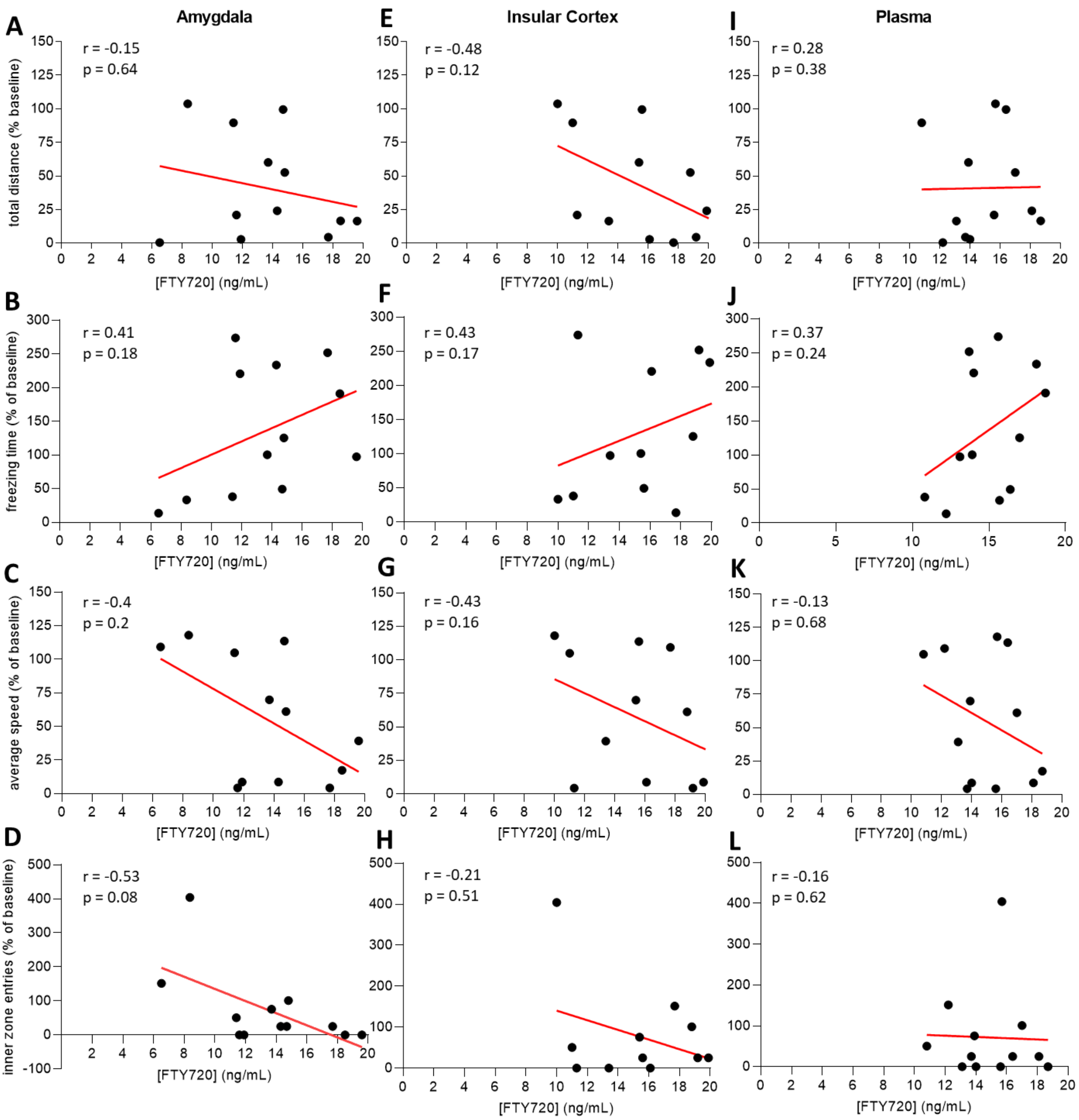

Supplementary Figure 1: Correlation between anxiety behavior assessed in the OF test and FTY720 concentrations in the AM, IC and plasma.

Relationship between the total distance traveled, freezing time, average speed as well as amount of inner zone entries (% of baseline) assessed in the OF test and the FTY720 concentrations (ng/ml) in the amygdala (A-D), the insular cortex (E-H) and plasma (I-L). Rats (n = 12) were injected three times every 72 h with 1 mg/kg bw FTY720 and tissue samples

were collected after behavioral testing. For each graph, a linear trend line, the correlation coefficient r and the p-value are indicated

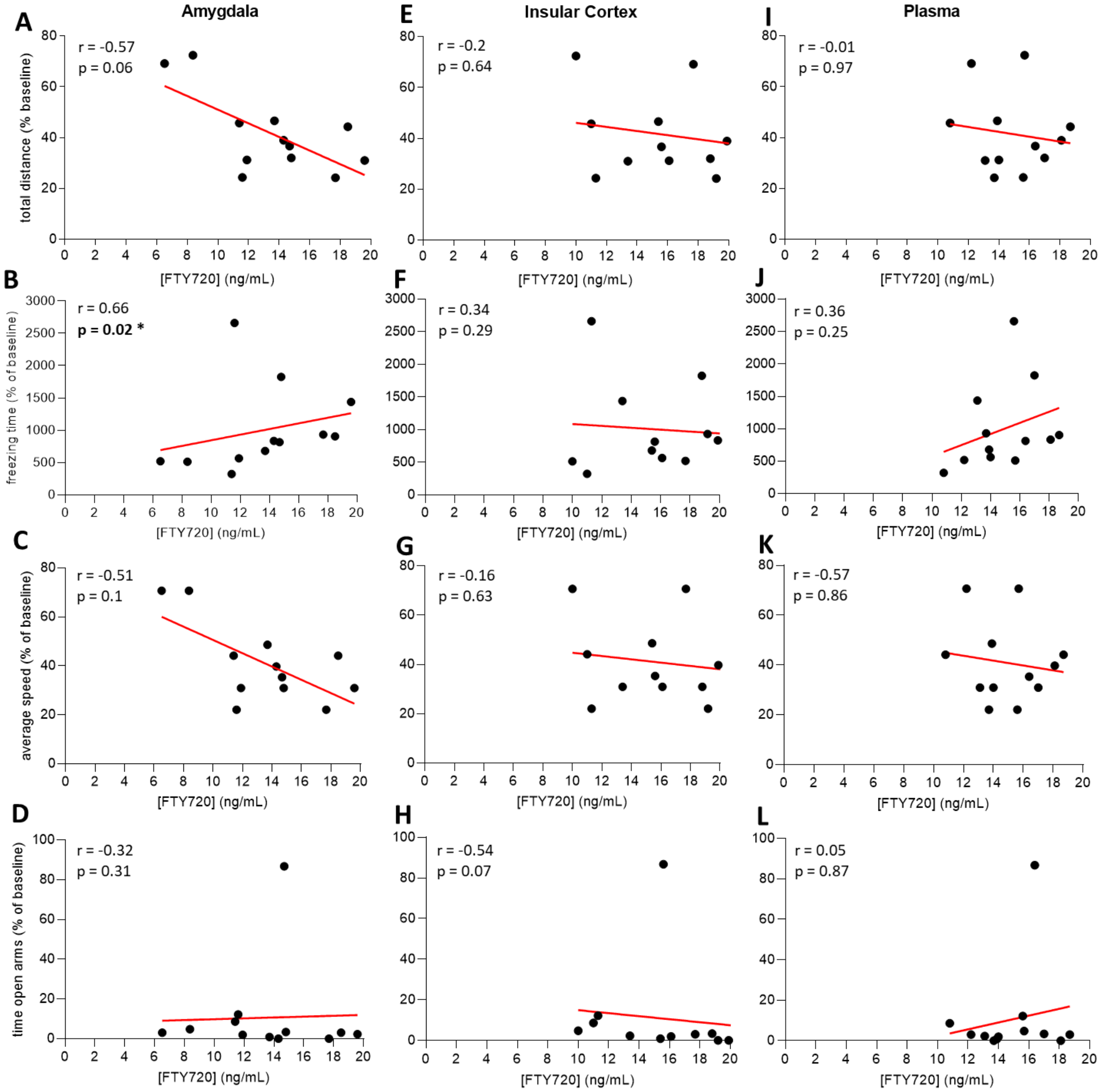

Supplementary Figure 2: Correlation between anxiety behavior assessed in the EPM test and FTY720 concentrations in the AM, IC and plasma.

Relationship between the total distance traveled, freezing time, average speed as well as time spent in the open arms (% of baseline) assessed in the EPM test and the FTY720 concentrations (ng/ml) in the amygdala (A-D), the insular cortex (E-H) and plasma (I-L). Rats (n = 12) were injected three times every 72 h with 1 mg/kg bw FTY720 and tissue samples

were collected after behavioral testing. For each graph, a linear trend line, the correlation coefficient r and the p-value are indicated. Asterisks represent a statistically significant correlation between both variables (Spearman´s correlation analysis; *p < 0.05).

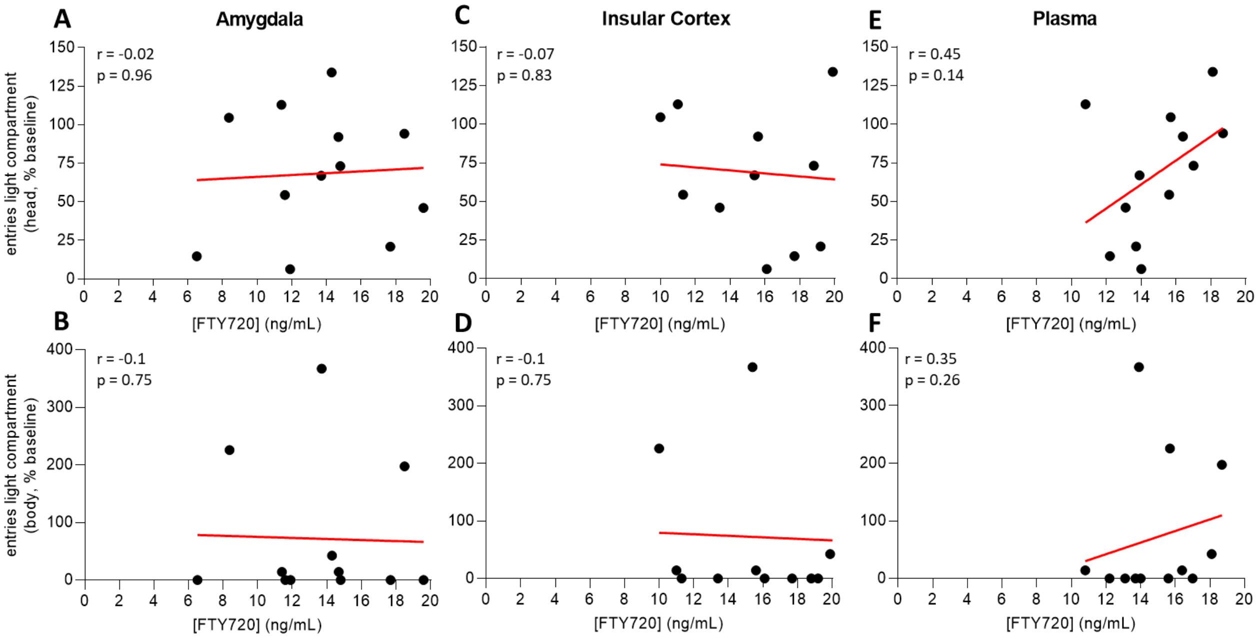

Supplementary Figure 3: Correlation between anxiety behavior assessed in the dark/light test and FTY720 concentrations in the AM, IC and plasma.

Relationship between the amount of times rats entered the light compartment with their head or with their whole body (% of baseline) assessed in the dark/light test and the FTY720 concentrations (ng/ml) in the amygdala (A-B), the insular cortex (C-D) and plasma (E-F). Rats (n = 12) were injected three times every 72 h with 1 mg/kg bw FTY720 and tissue samples were collected after behavioral testing. For each graph, a linear trend line, the correlation coefficient r and the p-value are indicated.

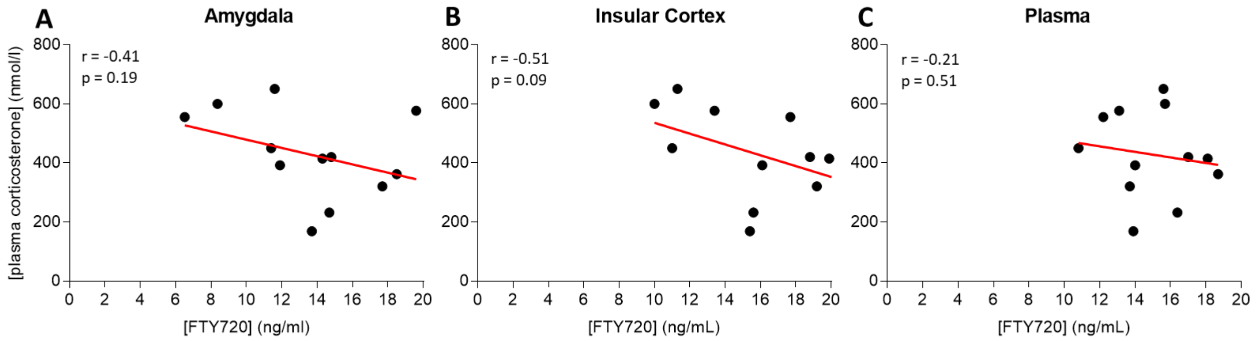

Supplementary Figure 4: Correlation between plasma corticosterone level and FTY720 concentrations in the AM, IC and plasma.

Relationship between plasma corticosterone concentrations (nmol/l) and the FTY720 concentrations (ng/ml) in the amygdala (A), the insular cortex (B) and plasma (C). Rats (n = 12) were injected three times every 72 h with 1 mg/kg bw FTY720 and tissue samples were collected after behavioral testing. For each graph, a linear trend line, the correlation coefficient r and the p-value are indicated.
